# Red blood cells morphology and morphometry in adult, senior, and geriatricians dogs by optical and scanning electron microscopy

**DOI:** 10.3389/fvets.2022.998438

**Published:** 2022-11-10

**Authors:** Ana Luisa Montoya-Navarrete, Alma Lilián Guerrero-Barrera, Teódulo Quezada-Tristán, Arturo G. Valdivia-Flores, María J. Cano-Rábano

**Affiliations:** ^1^Morphology Department, Basic Sciences Center, Autonomous University of Aguascalientes, Aguascalientes, Mexico; ^2^Veterinary Sciences Department, Agricultural Sciences Center, Autonomous University of Aguascalientes, Aguascalientes, Mexico; ^3^Department of Veterinary Medicine, Surgery and Anatomy, Faculty of Veterinary Medicine, University of León, León, Spain

**Keywords:** red blood cells morphology, optical microscopy, scanning electron microscopy, adult dogs, senior dogs, geriatric dogs

## Abstract

Red blood cells (RBC) morphologic evaluation through microscopy optical (OM) and SEM, provides information to forecast, evaluate, and monitor the functioning of many organs. Factors, such aging and diseases affect RBC morphology in both, human and animals. SEM is useful to evaluate RBC morphology, although its use in diagnosis and evaluation in dogs is limited, due to the availability and cost. The aim of this research was to assess the normal RBC morphology in adult, senior and geriatrician dogs, clinically healthy by OM and SEM. In addition to evaluating the age effect, sex, body size, and their interaction on erythrocyte morphometry. To carry out the research 152 blood samples were evaluated from dogs of different sexes and body sizes (small, medium, and large). Three groups were made based on dogs age: group I adults (1–7.9 years old), group II senior (8–11.9 years old), and group III geriatricians (>12 years old). Erythrocyte parameters were evaluated by OM (diameter, height, and axial ratio). Per each dog, the parameters of 20 erythrocytes were measured. A total of 2,600 cells were scanned with the AmScope™ Software scale. In addition, the RBC morphology was evaluated by SEM. Statistical analyses used analysis of variance and a general linear model, which allows the comparison of multiple factors at two or more levels (*p* < 0.05). The results of this study showed that diameter and height were lower in adult dogs than in senior and geriatrician dogs (*p* < 0.05). Whereas, sex, body size, and the interaction did not show a significant effect (*p* > 0.05). Additionally, some images of anisocytosis, polychromasia, and poikilocytosis (echinocytes, acanthocytes, codocytes, spherocytes, stomatocytes, dacryocytes quatrefoil, and elliptocytes) were obtained by OM and SEM. Our study provides information about the morphological and morphometry alterations of adult, senior, and geriatrician dogs RBC. This work contributes to future investigations and the diagnosing diseases, where it is necessary to evaluate the morphology of RBC.

## Introduction

Red blood cells (RBC) are the most abundant blood cells in the organism, delivering oxygen to body tissues. The count of these vital cells is often the first step done in analyzing a patient's pathological condition (1, 2). RBC are anucleated, and in the shape of biconcave discs with central pallor called discocytes (3). Poikilocytosis is the term used for abnormal shaped RBC in the blood (4). Assessing alterations in red blood cell morphology provides important information that can help to establish a differential diagnosis of diseases in both humans and animals (5–8). In addition to diseases, other physiological factors can cause changes in erythrocyte morphology, such as aging (9, 10).

The light microscope has been the most frequent method for the evaluation of the RBC morphology because is an important tool in the detection of diseases in geriatric patients, as well as a quality control measure for automated hematology equipment and as a rapid evaluation of patients in veterinary emergencies (11–13). However, the advancement of technology allows us to explore other tools that may be alternatives to assess the RBC morphology in a field of scientific research, as scanning electron microscopy (SEM) is widely used to evaluate the morphological properties of erythrocytes in different physiological conditions human (8, 14–16).

The morphological evaluation of RBC through SEM has been applied mainly in human medicine, in patients with inflammatory diseases, cardiovascular, related to aging, and diabetes (17–19). Some of these diseases can also be present in dogs, especially in aging dogs (7, 20–22). Thus, over the years the dog has been used as an experimental model in the advancement of some diseases that also occur in humans (22–25).

For this reason, it is important to explore other microscopy tools in the field of veterinary research that can assist in the diagnosis and monitoring of diseases in both animals and humans. Besides, to our knowledge, it has not been determined erythrocyte parameters in aging dogs. Therefore, this study aimed to evaluate RBC morphology in the adult dogs (1–7.9 years old), senior dogs (8–11.9 years old), and geriatrician dogs (>12 years old) clinically healthy by OM and SEM. In addition to evaluating the effect of age, sex, body size, and their interaction on erythrocyte morphometry through parameters of RBC (diameter, height, and axial ratio).

## Materials and methods

### Population

This study was carried out in compliance with the provisions established in the Ethics Regulations for the Use of Animals in Teaching and Research at the Autonomous University of Aguascalientes (CEADI-UAA) Code: DI-PL-NO-37 (26). A non-experimental transverse design was used (27). We selected 152 healthy dogs of different sexes and sizes classified by age: group I 49 adults (1–7.9 years old), group II 51 seniors (8–11.9 years old), and group III 52 geriatricians (>12 years old) (28). The breeds were grouped according to their body size: small-sized (< 9.5 kg), medium-sized (9.5–22.7 kg), and large-sized dogs (>22.7–54.5 kg) (29).

Animals were assessed as healthy on the basis of a complete physical examination and history. The collection of the medical history and the physical examination were carried out by a veterinarian who specializes in clinical medicine of small species at the Parasitology Laboratory of the Center for Agricultural Sciences of the Autonomous University of Aguascalientes and private clinics. A questionnaire (Supplementary Appendix 1) related to health, living environment, activity, behavioral changes, nutrition, vaccination, parasite control, and medical history was administered (30, 31). The questionnaire was reviewed with the owner, and additional questions were asked if required. During this time, the dog was allowed to freely explore the examination room. An animal information form in Supplementary Appendix 2 (documenting the date of birth, sex, breed, weight, body size, diet, reproductive status, and vital signs) was also obtained for each dog before inclusion in the study (32, 33). Moreover, a body condition score (BCS) was determined for each dog based on a 9-point scale (34).

All dogs fasted for 8–12 h before blood sampling, and water was offered at libitum. Non-fasted dogs, dogs on medication at the time of blood sampling, or when a significant illness was suspected based on history and observation were excluded from the study. Dogs needed to be free of medication for at least 2 months before inclusion. Preventive medication (deworming, vaccination) was allowed until 2 weeks before the consultation. Females were not pregnant, not lactating, and not in oestrus. All dogs selected for this study were privately owned (28–30). All owners signed an informed consent form (Supplementary Appendix 1). No type of anesthesia or sedation was used when taking the blood sample.

### Collection of blood samples

Samples of 500 μl of blood were collected using venipuncture jugular with vacuum tubes BD Vacutainer^®^ with EDTA K2, applied by aspersion (BD Franklin Lakes NJ USA) to perform the blood smear analysis.

### Optical microscopy

We performed the blood smears immediately after blood collection in the Diagnostic Pathology Laboratory of the Agricultural Sciences Center of the Autonomous University of Aguascalientes. Smears were fixed with methanol and stained with Wright stain. Subsequently, a coverslip with Entellan (Merck^®^, Darmstadt, Germany) and xylol (JT Baker^®^ Avantor, Matsonford Rd) was placed on them, and they were left to dry for 24 h (35). All smears were observed using a ZEISS optical microscope (Oberkochen, Germany). Images were obtained with an AmScope™ camera and software (USA).

#### Blood smear evaluation

The evaluation of the blood smear was divided into three parts: red series, white series, and platelets. A magnification of 100 × was used to evaluate the morphology of erythrocytes, leukocytes, and platelets (36). In the analysis of the red series were evaluated the size (anisocytosis, macrocytosis, and microcytosis), the color (polychromatophilic and hypochromic), the shape (non-specific and specific morphological changes), the distribution (stacks of coins or agglutination), and the presence of inclusion bodies or erythrocyte parasites (5, 37). The morphology of the leukocytes (white series) and platelets was evaluated (38).

#### Morphometry parameters of RBC

**Figure 1:**
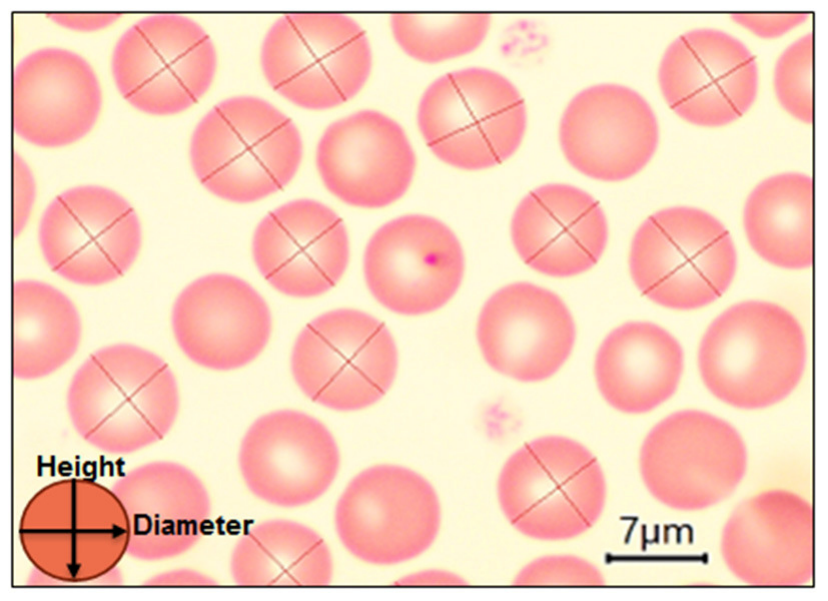
Blood smear from healthy dogs, showing positions for diameter and height calculations from optical micrographs using AmScope™ software. The diameter or major axis was measured in the erythrocytes and a perpendicular line was drawn in the center of the diameter to establish the height. Scale bar = 7 μm.

The erythrocyte parameters were determined from OM micrographs according to Loyola-Leyva et al. (14). Per each dog of study, 20 erythrocytes were evaluated. The longest axis, called the major axis or diameter, was measured in all RBC; a perpendicular line was drawn at the center of the major axis to establish the length of the minor axis or height (Figure 1). Then, the axial ratio was calculated by dividing the length of the major axis (diameter) by the length of the minor axis (height); a value of 1 represents a perfect circle (14). Therefore, 2,600 cells were scanned to assess the morphological changes. All samples were viewed with the Measurement scaler of the main menu of the AmScope™ software (USA).

### Scanning electron microscopy

A total of 3 ml of whole blood obtained by jugular venipuncture was collected and deposited in tubes with K2EDTA BD Vacutainer^®^, which contains the optimal amount of di-potassium EDTA applied by spray to anticoagulant the specified volume of blood (BD Franklin Lakes NJ USA). Once obtained the blood sample was centrifuged at 2,000 rpm for 10 min to separate the leukocyte layer that was deposited in a 1.5 ml Eppendorf tube, adding glutaraldehyde (JT Baker^®^ Avantor, Matsonford Rd), to fix them until further analysis. The sample, later, was centrifuged at 6,000 rpm for 1 min, obtaining only the precipitate by decanting the sample. Phosphate buffered saline (PBS) solution 1X (Merck^®^, Darmstadt Germany) was added to the precipitate, mixed, and allowed to rest for 5 min. This process was carried out three times; then it was added alcohol at 60%, letting the precipitate rest for 10 min. The sample was centrifuged again at 6,000 rpm for 1 min; this process was repeated with alcohol at 70, 80, 90, 96, and 100%. Lastly, 100% of pure acetone was added and fixed in a dark-bottomed aluminum cylinder to later be coated with gold particles in Desk II Denton vacuum equipment (Lawrence, Kansas), and finally observed in the scanning electron microscope JEOL JSM-5900LV (JEOL Solutions for innovation, Mexico City) (39, 40). Micrographs of the erythrocytes were obtained by SEM. Some electron microscopy images were photoshopped to add color to the blood cells.

### Statistical analysis

Statistical analysis was performed with Minitab 17 (Minitab Statistical Software, State College, PA); *p* < 0.05 was considered significant. We evaluated the distribution of the variables by examining the histograms and using a goodness of fit test (Anderson–Darling) (41). To determine if the variance of two or more groups was significantly different, we used a test of equality of variances with multiple comparisons and Levene's test. These methods are valid in non-normal distributions, while in normal distributions, the Bartlett test is used. All tests of variance used a confidence level of 95% (42).

Statistical analyses employed analysis of variance (ANOVA) with a general linear model (GLM), which allows the comparison of multiple factors at two or more levels (*p* < 0.05). Morphometric parameters of RBC: diameter, height, and axial ratio represent response variables (dependent), while age, sex, and body size represent factors (independent variables). The interaction between factors was also evaluated. When the data did not meet the normality assumptions and homoscedasticity, we performed a Box-Cox transformation using Minitab's optimal lambda (λ) with a confidence level of 95%. Subsequently, a multiple comparison method (Tukey) was performed with a confidence level of 95% (43).

Reference intervals for each of the morphometric variables (diameter, height, and axial ratio) were calculated using the software Reference Value Advisor (RefValAdvV.2.1, http://www.biostat.envt.fr/reference-value-advisor/) based on the recommendations of International Federation of Clinical Chemistry (IFCC) and the Clinical and Laboratory Standards Institute (CLSI) (44). This software detected the outliers with Tukey and Dixon tests, showing the distribution (dot plot and histograms) and QQ plot for visual inspection (30, 44).

## Results

A total of 152 blood samples were collected from dogs of different ages: group I adults (1–7.9 years old), group II seniors (8–11.9 years old), and group III geriatricians (>12 years old). Eighty-two dogs were female, and 66 dogs were male. All dogs were grouped according to their body size: 81 small dogs, 25 medium dogs, and 42 large dogs. A variety of small, medium and large dogs were represented in each age group. Nineteen blood samples were excluded: five samples due to the presence of clots, four samples with hemolysis and 10 samples from the statistical analysis because of underlying subclinical diseases. Therefore, only 129 blood samples from clinically healthy dogs were analyzed by light microscopy and 4 blood samples by scanning electron microscopy, as described in Table 1.

**Table 1:** Number of blood samples from clinically healthy dogs analyzed in this study by light microscopy and scanning electron microscopy.

	**Adult dogs**	**Senior dogs**	**Geriatric dogs**	**Total**
Optical microscopy (OM)	*n* = 44	*n* = 41	*n* = 44	*n* = 129
Scanning electron microscopy (SEM)	*n* = 2	*n* = 1	*n* = 1	*n* = 4
Excluded samples	*n* = 3	*n* = 9	*n* = 7	*n* = 19
Total	*n* = 49	*n* = 51	*n* = 52	*n* = 152

### Optical microscopy

**Figure 2:**
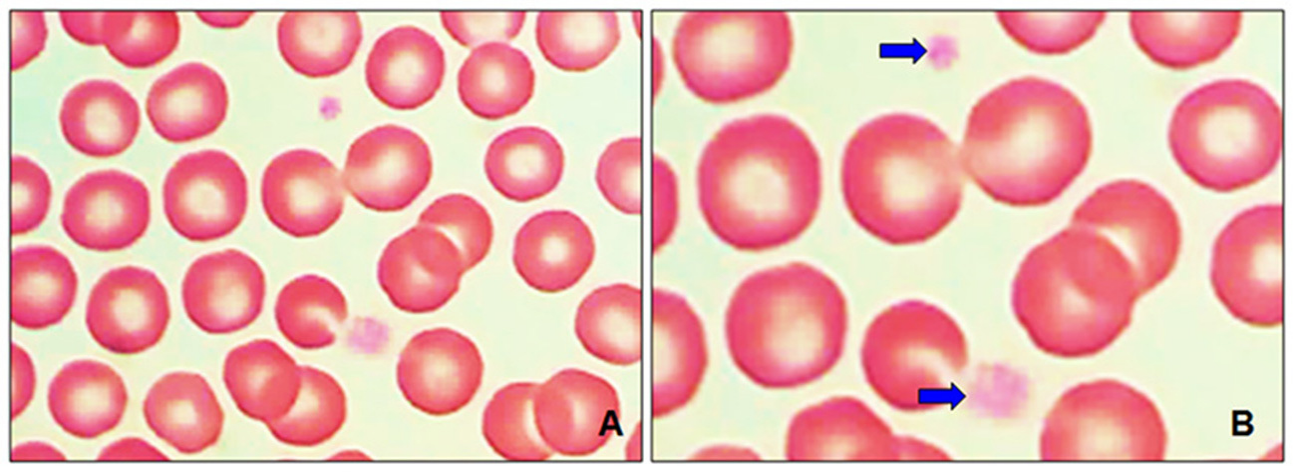
Light microscopy smears healthy dogs at 100 ×. **(A)** The normal morphology of red blood cells (RBC) shows a pale central area that represents 1/3 of their diameter approximately. **(B)** Platelets with a normal central cluster of small pink or purple granules in the cytoplasm (blue arrows).

**Figure 3:**
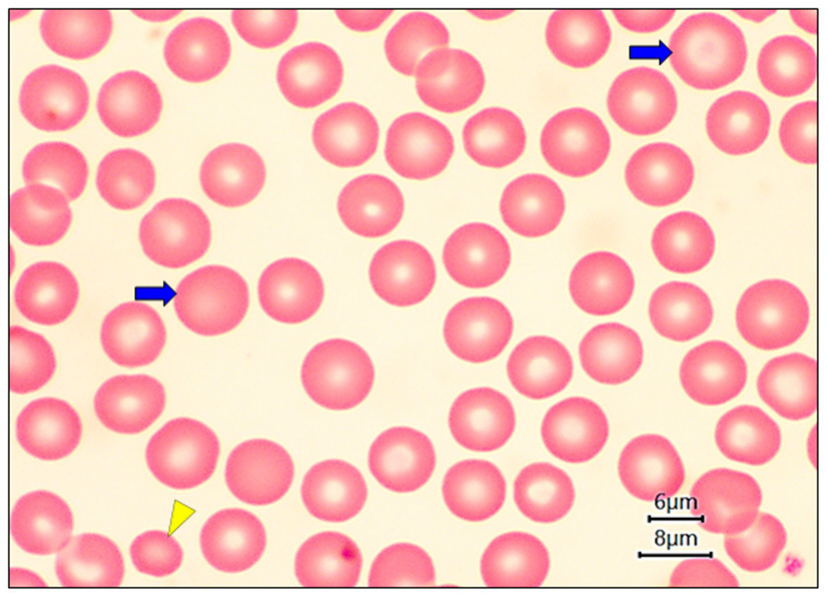
Light microscopy smears healthy dogs at 100 ×. The presence of red blood cells of the abnormal size called anisocytosis (size variation) is shown, and a microcyte is observed in the lower-left corner (yellow arrowhead) and macrocytes (blue arrows). Scale bars = 6 and 8 μm.

In this research were obtained images of the blood smears by light microscopy. Figures 2A,B show the normal RBC morphology and small platelets with pale gray to light blue cytoplasm with normal granule content. On evaluation regarding erythrocyte morphology, anisocytosis (size variation) was recorded in the 88% of samples (Figure 3), polychromasia (polychromatophilic erythrocytes) y poikilocytosis (abnormal form). Abnormal forms of erythrocytes were specifically classified.

Table 2 shows the frequency of each of the variables such as the size, color, shape, and inclusion bodies of the erythrocytes that were observed in the blood smear by optical microscopy in each of the groups of dogs (adults, seniors, and geriatricians), where the absolute frequency, cumulative frequency, relative frequency, cumulative relative frequency, frequency in percentage, and cumulative frequency in percentage are described.

**Table 2:** Frequency analysis of the variables evaluated in the blood smear of a population of dogs (*n* = 129) of different ages: adults (*n* = 44), seniors (*n* = 41), and geriatrics (*n* = 44).

**Variable**	**Finding**	**Group**	**Absolute frequency**	**Accumulated frequency**	**Relative frequency**	**Cumulative relative frequency**	**Percentage frequency %**	**Cumulative percentage frequency %**
Size	Anisocytosis	Adult	37	37	0.29	0.29	29	29
		Senior	34	71	0.26	0.55	26	55
		Geriatric	43	114	0.33	0.88	33	88
	Macrocytosis	Adult	2	2	0.02	0.02	2	2
		Senior	5	7	0.04	0.05	4	5
		Geriatric	8	15	0.06	0.12	6	12
Color	Polychromatophilic	Adult	2	2	0.02	0.02	2	2
		Senior	8	10	0.06	0.08	6	8
		Geriatric	11	21	0.09	0.16	9	16
Shape	Echinocytes	Adult	11	11	0.09	0.09	9	9
		Senior	10	21	0.08	0.16	8	16
		Geriatric	12	33	0.09	0.26	9	26
	Acanthocytes	Adult	32	32	0.25	0.25	25	25
		Senior	32	64	0.25	0.50	25	50
		Geriatric	35	99	0.27	0.77	27	77
	Codocytes	Adult	18	18	0.14	0.14	14	14
		Senior	16	34	0.12	0.26	12	26
		Geriatric	13	47	0.10	0.36	10	36
	Spherocytes	Adult	5	5	0.04	0.04	4	4
		Senior	1	6	0.01	0.05	1	5
		Geriatric	8	14	0.06	0.11	6	11
	Stomatocytes	Adult	1	1	0.01	0.01	1	1
		Senior	1	2	0.01	0.02	1	2
		Geriatric	1	3	0.01	0.02	1	2
	Dacryocytes	Adult	14	14	0.11	0.11	11	11
		Senior	14	28	0.11	0.22	11	22
		Geriatric	15	43	0.12	0.33	12	33
	Quatrefoil	Adult	6	6	0.05	0.05	5	5
		Senior	3	9	0.02	0.07	2	7
		Geriatric	4	13	0.03	0.10	3	10
	Elliptocytes	Adult	1	1	0.01	0.01	1	1
		Senior	1	2	0.01	0.02	1	2
		Geriatric	1	3	0.01	0.02	1	2
Inclusion bodies	Erythroblasts	Adult	1	1	0.01	0.01	1	1
		Senior	4	5	0.03	0.04	3	4
		Geriatric	4	9	0.03	0.07	3	7

**Figure 4:**
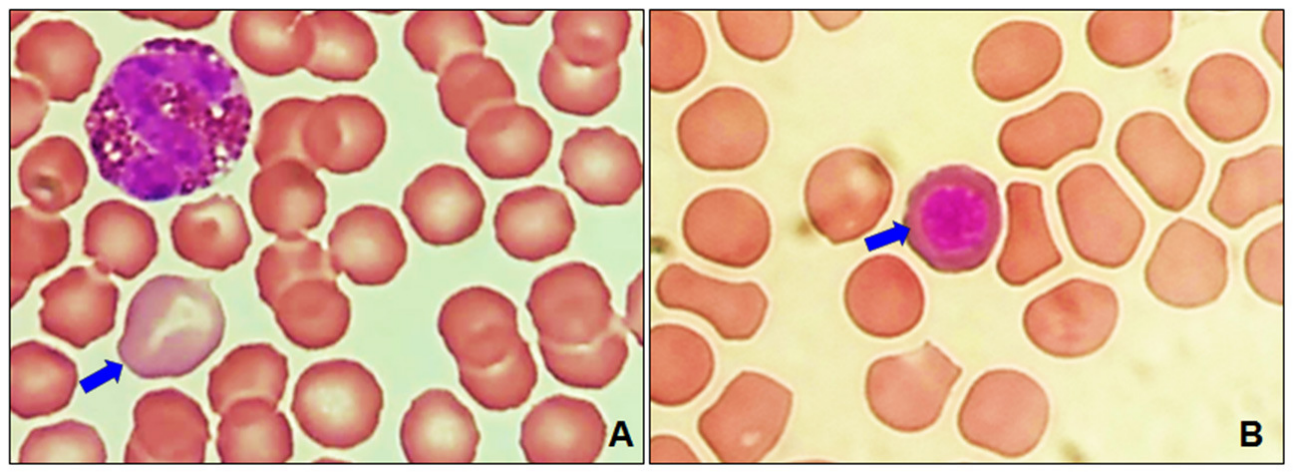
Optical micrographs at 100 × of senior and geriatric dogs. **(A)** Polychromatophilic erythrocytes have a bluish cytoplasm and are larger than a normal red blood cell (blue arrow). **(B)** Erythroblasts are nucleated precursor cells of erythrocytes (blue arrow).

**Figure 5:**
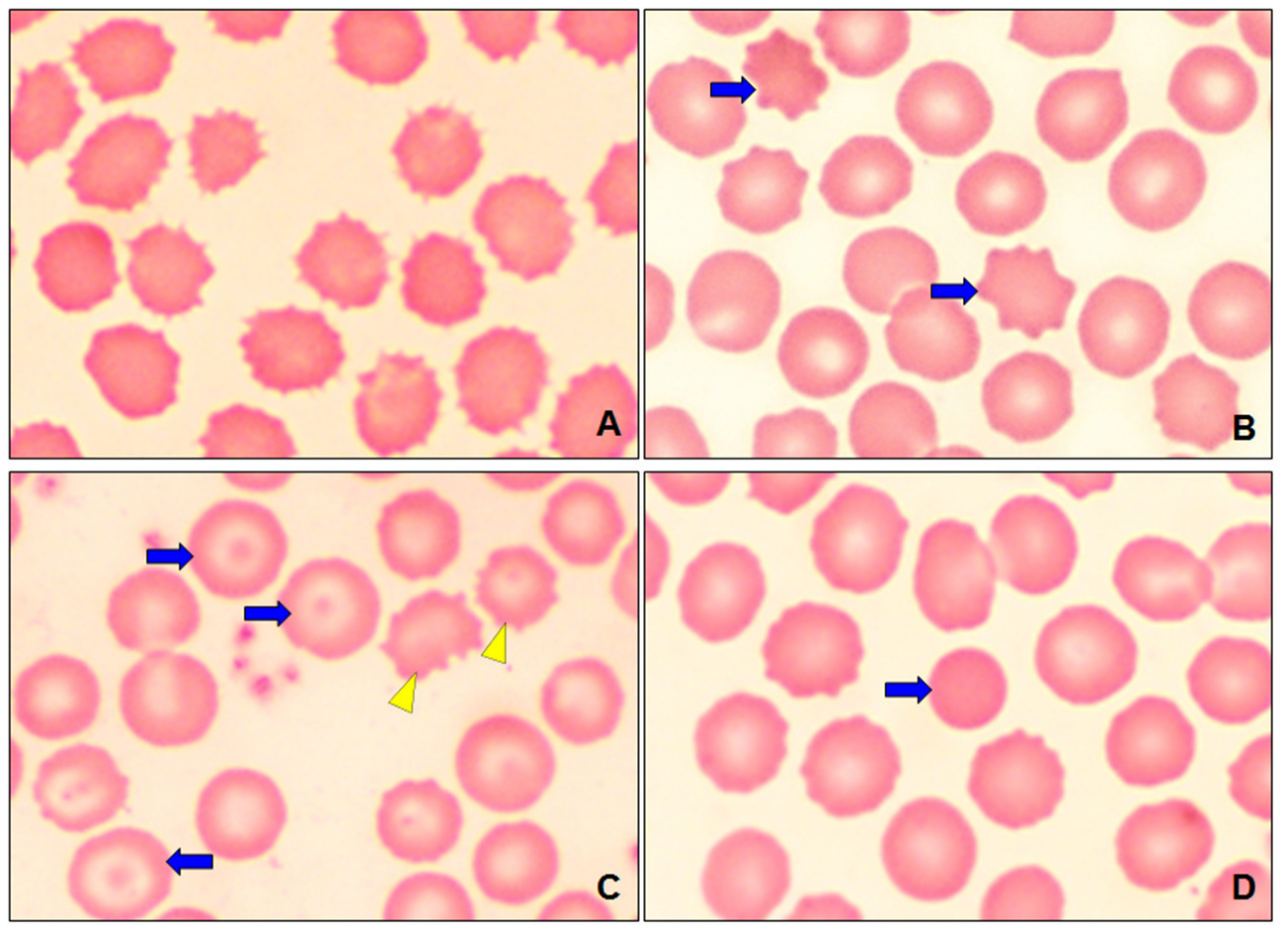
Optical micrographs at 100 × of dogs, showing the poikilocytosis (abnormal forms of erythrocytes). **(A)** Echinocytes. **(B)** Acantocytes (blue arrows). **(C)** Codocytes or target cells (blue arrows) and acanthocytes (yellow arrowhead). **(D)** Spherocyte (blue arrow).

**Figure 6:**
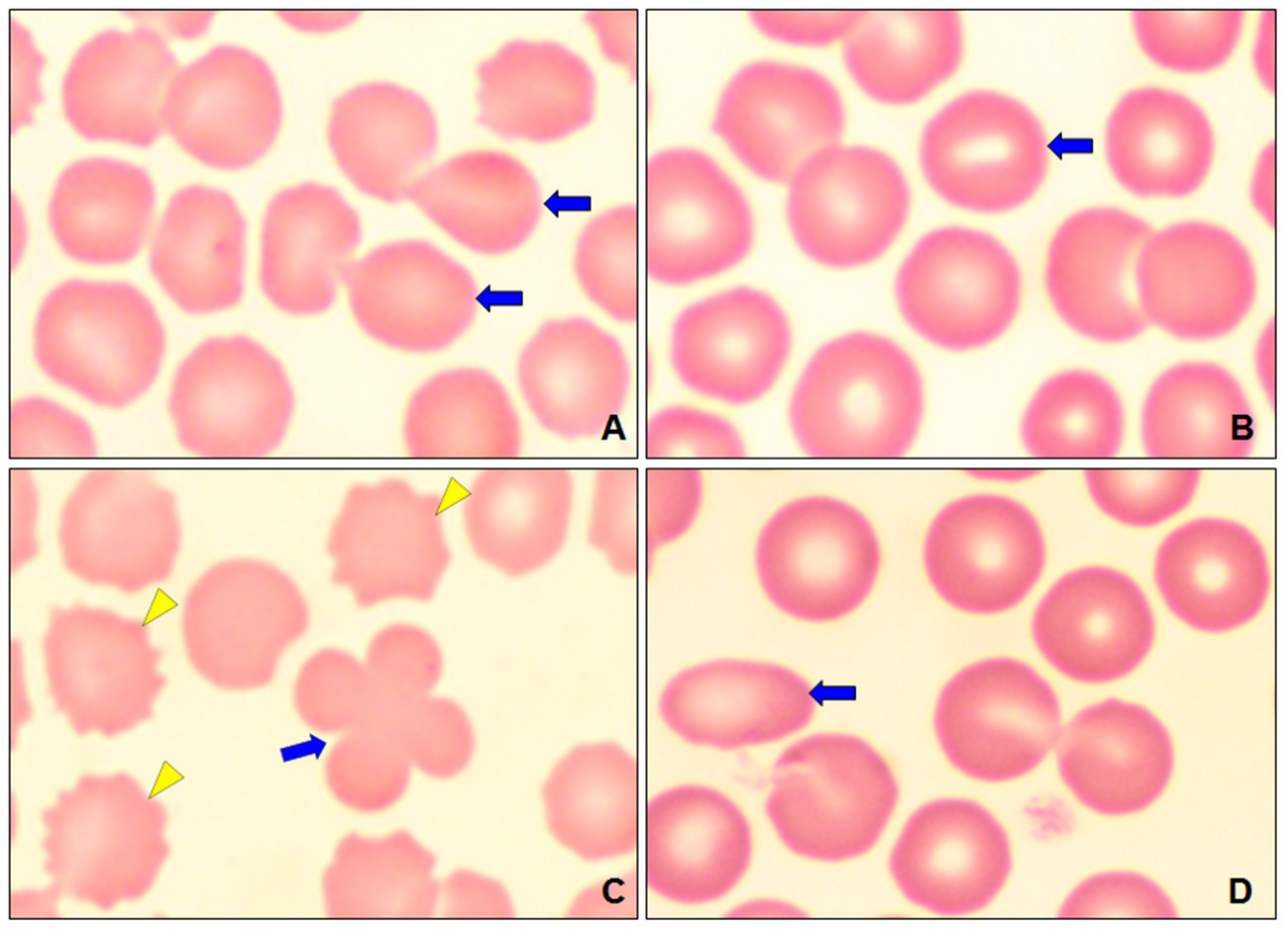
Optical micrographs at 100 × of dogs, showing the poikilocytosis (abnormal forms of erythrocytes). **(A)** Dacrocytes (blue arrow). **(B)** Stomatocytes (blue arrow). **(C)** Cross-shaped or quatrefoil erythrocyte (blue arrow) and acanthocytes (yellow arrowhead). **(D)** Elliptocytes (blue arrow).

A presence of polychromasia (Figure 4A) was identified in geriatric dogs, accompanied by macrocytes (Figure 3) and erythroblasts (Figure 4B). While in poikilocytosis the presence of abnormal erythrocyte forms was indistinct. However, three abnormal erythrocyte forms were observed most frequently (Table 1): acanthocytes (77%), codocytes (36%), and dacryocytes (34%). Figure 5A shows echinocytes with small spicules evenly spaced on their surface, a detail that differentiates them from acanthocytes (Figures 5B,C) that have irregular projections. Figure 5C also shows target-shaped erythrocytes (target cells or codocytes). Figure 5D shows small erythrocytes, without central paleness and dense staining (spherocytes). Teardrop-shaped erythrocytes known as dacryocytes were observed in Figure 6A. Erythrocytes with oval or elongated central pallor are described as stomatocytes in Figure 6B. Quatrefoils are cells in the form of a cross (Figure 6C). Elongated or elliptical red blood cells (elliptocytes) are shown in Figure 6D. Inclusion bodies indicative of erythropoiesis such as erythroblasts were detected in 7% of the samples analyzed (Figure 4B). No distribution abnormalities (pseudo phenomenon rouleaux) were observed, or erythrocyte parasites were found.

**Figure 7:**
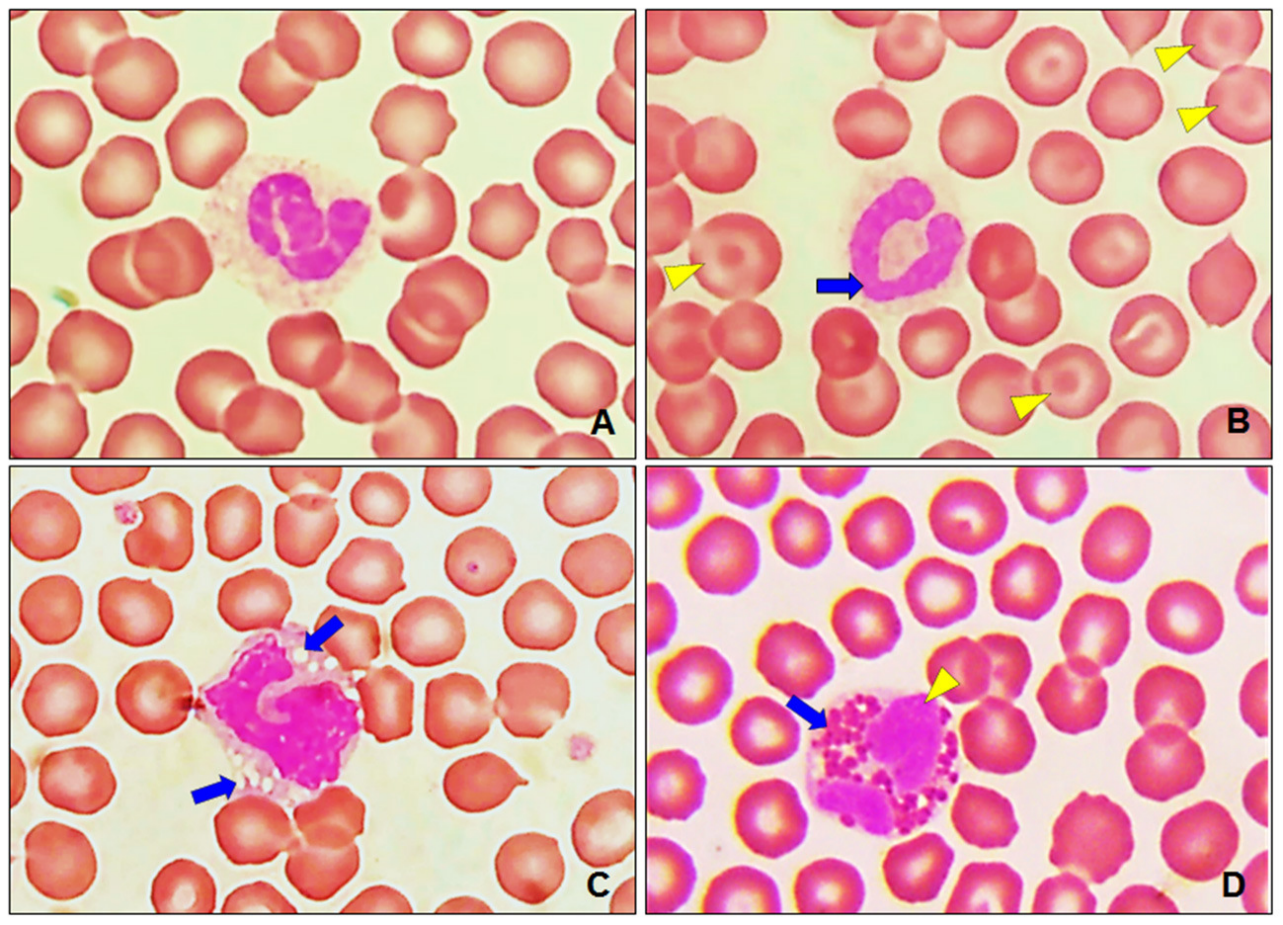
Leukocytes in healthy dogs. **(A)** Segmented neutrophil with an elongated and irregularly lobed nucleus. **(B)** Band neutrophil with a curved nucleus and slight strangulation (blue arrow), codocytes (yellow arrowhead). **(C)** Monocyte with the presence of vacuoles in the cytoplasm (blue arrow), optical microscopy. **(D)** Eosinophil with orange-red granules (blue arrow) and a segmented nucleus (yellow arrowhead). Optical microscopy at 100 ×.

In Figure 7A, it can observe the morphology of a segmented neutrophil that shows a lobed nucleus and cytoplasm with slightly eosinophilic granulation, while Figure 7B shows a band neutrophil with a curved nucleus and slight strangulation, noticing that its cytoplasm shows a slightly eosinophilic granulation as well. Additionally, Figure 7C shows the morphology of a monocyte with the presence of vacuoles and gray-blue cytoplasm with an indented nucleus. The monocyte nucleus varies greatly and can take any shape from round, indented, coiled, or kidney-shaped. The presence of vacuoles of variable size in the cytoplasm is characteristic. On the other hand, in Figure 7D, eosinophils were identified, presenting red-orange granules that are distinctive in a pale blue cytoplasm. No abnormalities were observed in leukocyte and platelet morphology.

**Figure 8:**
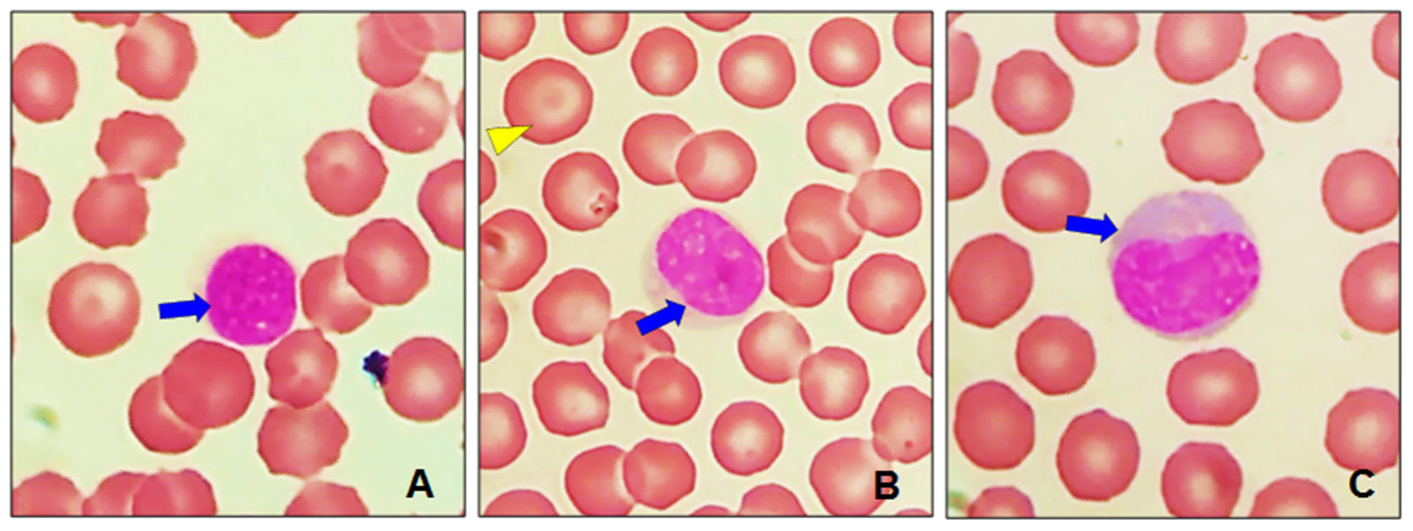
Leukocytes in healthy dogs. **(A)** Small lymphocyte with its round nucleus strongly dyed (blue arrow). **(B)** Small elongated and whitish area, close to the nucleus, typical of lymphocytes (blue arrow), codocyte (yellow arrowhead). **(C)** Large lymphocyte, with more abundant cytoplasm (blue arrow) and less dense nuclear chromatin. Optical microscopy at 100 ×.

Normal lymphocyte morphology was identified in the blood of dogs by light microscopy. Figure 8A shows a small lymphocyte, its nucleus can be round or oval, sometimes slightly indented, or strongly stained. Also, in Figure 8B the cytoplasm of the lymphocytes is sparse, and light blue in color. Figure 8C shows a large lymphocyte, its cytoplasm is more abundant ranging from mild to moderate basophilia.

### Morphometry parameters of RBC

Table 3 shows the mean, standard deviation, median, interquartile range, and the significant difference with a 95% confidence interval; the lower limit and upper limit were determined with a confidence interval of 90% from each of the morphometric parameters of RBC. The effect of age, sex, and body size on erythrocyte parameters (diameter, height, and axial ratio) were assessed. The following statistical data are reported in the results: the significance level (*p*), mean (*M*), and standard deviation (*SD*).

**Table 3:** Parameters of red blood cells (RBC) evaluated by optical microscopy (OM).

	**(I) Adults**		**(II) Senior**		**(III) Geriatric**		
	**1–7.9 year old**		**8–11.9 year old**		>**12 year old**		
	***n*** = **44**		***n*** = **41**		***n*** = **44**		
**Variables**	**Mean (SD)**	**LL (90% CI)**	**Mean (SD)**	**LL (90% CI)**	**Mean (SD)**	**LL (90% CI)**	***p* value**
	**Median (IQR)**	**UL (90% CI)**	**Median (IQR)**	**UL (90% CI)**	**Median (IQR)**	**UL (90% CI)**	
Diameter (μm)	7.56 (0.44)^a^	6.7 (6.7–6.8)	7.67 (0.49)^b^	6.8 (6.7–6.9)	7.72 (0.45)^b^	6.9 (6.8–6.9)	0.00
	7.54 (0.61)	8.5 (8.4–8.6)	7.65 (0.66)	8.7 (8.6–8.7)	7.70 (0.63)	8.7 (8.6–8.7)	
Height (μm)	7.18 (0.42)^a^	6.4 (6.3–6.4)	7.29 (0.45)^b^	6.5 (6.4–6.5)	7.35 (0.44)^c^	6.5 (6.5–6.6)	0.00
	7.16 (0.54)	8.0 (8.0–8.1)	7.28 (0.58)	8.2 (8.1–8.3)	7.35 (0.62)	8.2 (8.2–8.3)	
Axial ratio	1.05 (0.04)^a^	1.0 (1.0–1.0)	1.05 (0.04)^a^	1.0 (1.0–1.0)	1.05 (0.04)^a^	1.0 (1.0–1.0)	0.56
	1.04 (0.05)	1.2 (1.1–1.2)	1.04 (0.06)	1.2 (1.1–1.2)	1.04 (0.06)	1.2 (1.2–1.2)	

CI, Confidence interval; IQR, Interquartile rage; LL, Lower limit; SD, standard deviation; UL, Upper limit. ^a, b, c^Groups that do not share the same letter are significantly different (*p* < 0.05).

**Figure 9:**
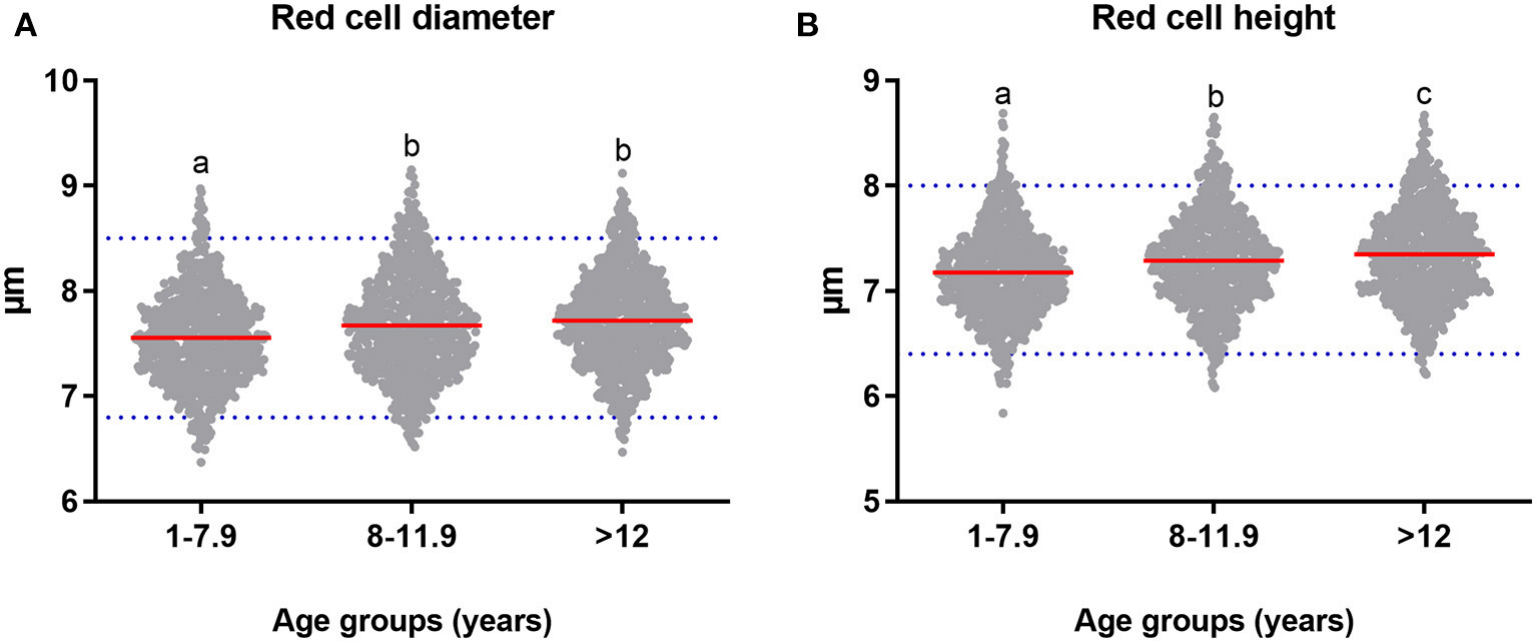
The measures of erythrocyte morphometric parameters at different stages of the dog's life. Comparison of the means (red line) **(A)** diameter and **(B)** height between groups of clinically healthy dogs of different ages: adults (*n* = 44 dogs of 1–7.9 years old), seniors (*n* = 41 dogs of 8–11.9 years old), and geriatrics (*n* = 44 dogs of >12 years old). Horizontal blue dotted lines represent the RIs for adult dogs with a 90% CI (see Table 3). Means that do not share a letter are significantly different (*p* < 0.05).

Age had a significant effect on the diameter (*p* = 0.00). The average red cell diameter in adult dogs from 1 to 7.9 years old was 7.56 μm (*SD* = 0.44), which was significantly lower than the diameter in seniors (*M* = 7.67 μm, *SD* = 0.49; *p* =0.004), and geriatric dogs (*M* = 7.72 μm, *SD* = 0.45; *p* = 0.00). Our research showed that diameter increased with age (Figure 9A). No significant effect of sex (*p* = 0.069), body size (*p* = 0.512), and interaction between age, sex, and body size (*p* = 0.238) was observed on the diameter.

Age had a significant effect on height (*p* = 0.00). Figure 9B shows that height in adult dogs (*M* = 7.18 μm, *SD* = 0.42) was significantly lower than the height of senior dogs (*M* = 7.29 μm, *SD* = 0.45; *p* = 0.002) and geriatric dogs (*M* = 7.35 μm, *SD* = 0.44, *p* = 0.00). Furthermore, height in senior dogs was significantly lower than height in geriatric dogs (*p* = 0.027). No effect of sex (*p* = 0.867), body size (*p* = 0.335), and interaction between age, sex, and body size (*p* = 0.414) on height was identified. No statistically significant effect of age (*p* = 0.56), sex (*p* = 0.662), body size (*p* = 0.078), and interaction between age, sex, and body size (*p* = 0.298) on the axial relationship was identified.

### Scanning electron microscopy

**Figure 10:**
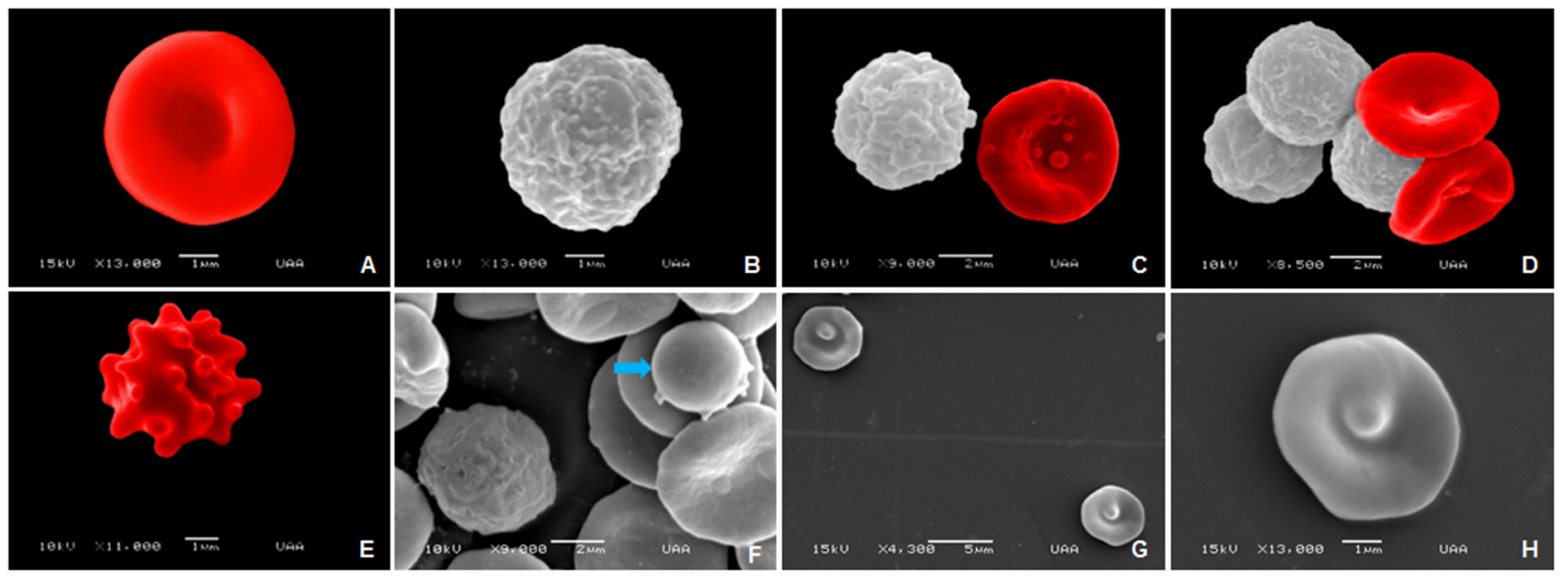
SEM images of normal and abnormal blood cells in adult dogs. **(A)** Erythrocyte. **(B)** Leukocyte. **(C)** Comparison of a leukocyte (left) and an erythrocyte (right). **(D)** Agglutination of erythrocytes and leukocytes. **(E)** Equinocyte. **(F)** Spherocyte (blue arrow). **(G,H)** Codocytes.

The following images were obtained through SEM and show the normal RBC morphology and leukocytes in adult, senior, and geriatric dogs. The Images of blood cells of adult dogs are shown in Figure 10, the normal RBC morphology is observed, with a characteristic smooth and concave surface (Figure 10A). In Figure 10B, the morphology of granulocytes (neutrophils) is seen which shows a rough surface. A comparison is also between the morphology and size of leukocytes and erythrocytes (Figures 10C,D). In addition, poikilocytosis was identified, as echinocytes, which are characterized by uniform protuberances (Figure 10E), smaller RBC known as spherocytes (Figure 10F), and codocytes, RBC with a densely dark center and surrounded by a pale halo around which an irregular dark band appears (Figures 10G,H).

**Figure 11:**
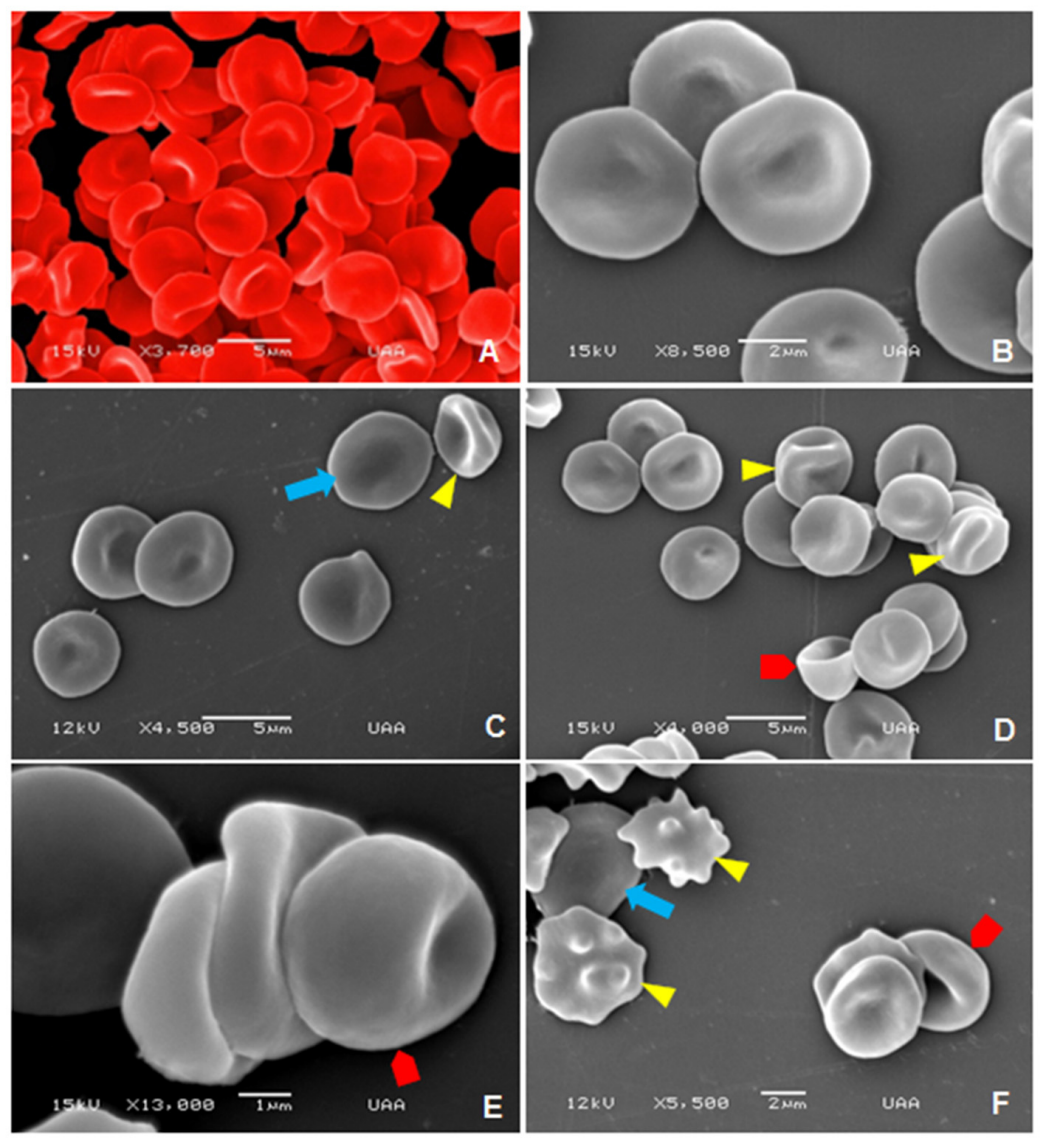
SEM images of normal and abnormal blood cells in senior dogs: **(A,B)** Normal erythrocyte morphology. **(C)** Leptocyte (blue arrow) and knizocyte (yellow arrowhead). **(D)** Stomatocyte (red pentagon arrow) and knizocytes (yellow arrowhead). **(E)** Stomatocyte (red pentagon arrow). **(F)** Leptocyte (blue arrow), equinocytes (yellow arrowhead), and stomatocyte (red pentagon arrow).

As for the senior dogs, in Figures 11A,B the normal morphology of the erythrocytes is observed. The abnormalities identified in this group were leptocytes, these cells are thin, generally large in diameter, and displays often hypochromic-appearing erythrocytes with increased membrane-to-volume ratios (Figures 11C,F), knizocytes mature RBC showing three concavities with two lighter areas “Pinch cells” (Figures 11C,D), stomatocytes, cells with one or two cavities, that is, a mouth-shaped cleft (Figures 11D–F) and echinocytes (Figure 11F).

**Figure 12:**
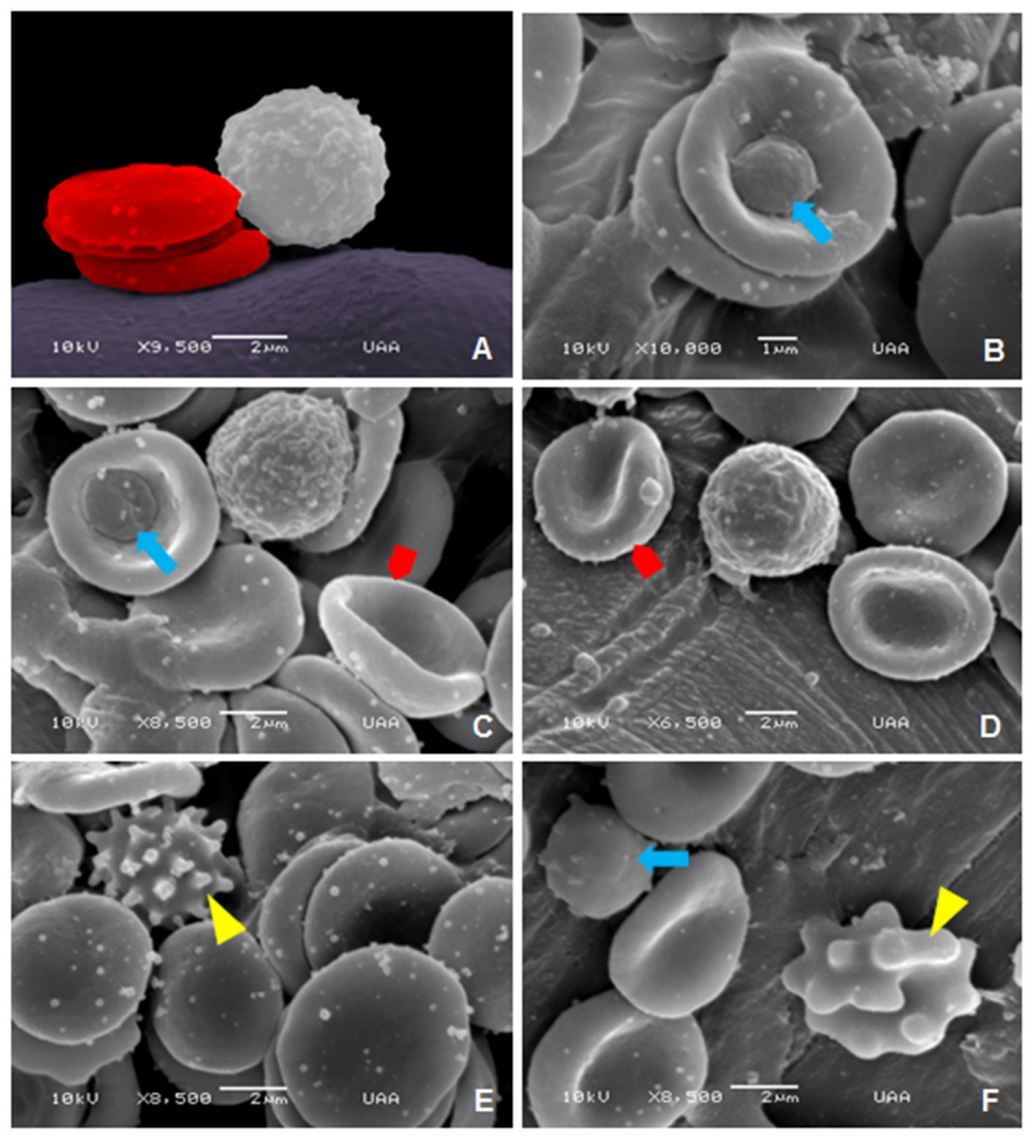
SEM images of normal and abnormal blood cells in geriatric dogs: **(A)** Normal morphology of erythrocytes and leukocytes. **(B,C)** Platelets (blue arrow). **(C,D)** Stomatocytes (red pentagon arrow). **(E,F)** Equinocytes (yellow arrowhead) and spherocytes (blue arrow).

Finally, Figure 12 shows the normal morphology of blood cells in geriatric dogs (Figures 12A,B). Additionally, platelets adhered to erythrocytes are shown (Figures 12B,C). In this group, the abnormalities identified in the erythrocytes were stomatocytes (Figures 12C,D), echinocytes (Figure 12E), and spherocytes (Figure 12F).

## Discussion

Meanwhile, in the evaluation of the optical microscopy of the red series, in terms of size and color, anisocytosis and polychromatophilia were observed. This can be observed under normal conditions. In particular, polychromatophilia can be identified in Wright-stained smears, but this observation must be < 1% (45). In addition, polychromasia was observed mainly in geriatric dogs in conjunction with macrocytes and erythroblasts. The loss of the nucleus of red blood cells (erythroblasts) gives rise to reticulocytes, larger red blood cells (macrocytes), and basophils (blue coloration) (46). Polychromatophilic erythrocytes are reticulocytes that stain bluish-red because of the combined presence of hemoglobin (red-staining) and ribosomes (blue-staining) (38). Polychromatophilia, whenever moderate, is a physiological phenomenon in bone marrow erythrocyte regeneration, which is generally accompanied by a significant number of macrocytes. Its slightly purple or violaceous appearance is determined by the high concentration of RNA in the aggregated reticulocytes and when this concentration is low it is indicative of the presence of stippled reticulocytes (47).

On the other hand, poikilocytosis like echinocytes, acanthocytes, codocytes, spherocytes, stomatocytes, dacryocytes, quatrefoil, elliptocytes, and leptocytes was observed in the OM and SEM micrographs. Three abnormal erythrocyte forms were observed most frequently: acanthocytes, codocytes, and dacryocytes. This is consistent with hematologic findings in dogs by other authors (48). Echinocytes are present in glomerulonephritis, uremia, lymphomas, alkalosis, and following the administration of furosemide and doxorubicin. Also, those may appear as artifacts if the collected blood volume is too low in relation to the EDTA content in the vial (49, 50). Acanthocytes in dogs appear mainly in chronic liver diseases, portosystemic shunt, disseminated intravascular coagulation (DIC), neoplasms, and nephropathies (glomerulonephritis) (51). Codocytes are also known as target cells. They occur in association with liver damage, kidney disease, iron deficiency anemia, disorders of the bile ducts, and spleen (37, 52, 53). Spherocytes, which are small cells without a central pallor, are characteristic of hereditary spherocytosis and autoimmune hemolytic anemia (54, 55). Stomatocytes result from red blood cell membrane defects found in hemolytic diseases and hereditary diseases (56, 57). Dacryocytes appear mainly in myeloproliferative disorders, hypersplenism, and glomerulonephritis. They are observed as tear-shaped red blood cells due to damage to the cell membrane, passing through the narrow medullary or splenic sinusoids (46, 51). Quatrefoil is a cell in the form of a cross, associated with the aging of dogs as described in a 2014 study (9). However, in this study, the presence of quatrefoil erythrocytes was independent of age.

Another important finding is the presence of elliptocytes and leptocytes. A study conducted on humans with autistic disorder and non-autistic neurodevelopmental disorders (NA-NDD), In particular observed, that abnormally shaped erythrocytes in the blood samples from autistic subjects predominantly featured elliptocytes. While, the NA-NDDs group showed mixed abnormally shaped RBCs, without a predominant erythrocyte shape phenotype, although a slight prevalence of leptocytes was detectable (58). In parallel to the recent advances in canine behavior research, dogs have also been proposed as a model for many human neuropsychiatric conditions, including autism spectrum disorder (24). Elliptocytes are erythrocytes elliptical or oval in shape, they are generally flat rather than biconcave (38). Hereditary elliptocytosis has been reported in a dog due to a mutant β-spectrin (59). Leptocytes are a thin, large diameter erythrocyte with increased surface area and normal cell volume (e.g., target cells and transverse folded cells) that readily distorts and gives a pinkish hue to the buffy coat layer. They are found in chronically diseased animals and in hepatic disease, obstructive jaundice, regenerative anemia, and iron deficiency anemia. Leptocytes tend to become knizocytes: a triconcave erythrocyte seen in hemolytic anemias in dogs and humans (60).

Morphological changes in RBC have been associated with an increased risk of cardiovascular diseases in human medicine (19). In inflammatory conditions, in presence of hydroxyl radicals, some researchers have found that RBC lose their discoid form (61). Some recent observations based on different types of microscopy concluded that iron has important effects on the morphology and deformability of RBC (62). Nonetheless, these important contributions have been applied to human medicine while the information about the use of scanning electron microscopy as a diagnostic tool in veterinary medicine is quite limited, due to the availability and cost of the technique.

RBC diameter evaluated in this study from the OM micrographs obtained a mean in adult dogs of 7.56 μm lower than of diameter of senior (7.67 μm) and geriatric dogs (7.72 μm). These results show an effect of age on the erythrocyte diameter. Some authors report that dogs' erythrocyte diameter varies from 6 to 8 μm (45, 63). An effect of age on erythrocyte height was also observed, increasing with age (Table 3). These results can be related to the presence of polychromasia described in this study. Polychromasia, as mentioned above, appears in ~1% of healthy dogs. However, higher proportions may indicate increased erythropoiesis, blood loss, hemolytic disease, or remission phases of anemia (46). No statistically significant differences were observed in RBC axial ratio. An effect of sex, body size, and the interaction (age, sex, and body size) on diameter, height, and the axial ratio was also not observed. Other authors have shown that breed and age have a significant effect on RBC morphometry (64, 65).

## Conclusion

It is important to evaluate the erythrocyte morphology and morphometry, especially in aged dogs, since this undoubtedly favors the diagnosis and prognosis of the aged patient. Optical microscopy is the tool par excellence to evaluate the blood smear, however, it is important to know other visual tools. Some diseases in humans also occur in dogs, therefore, the latter could be used in future research to help evaluate the morphology and RBC parameters by means of optical microscopy and scanning electron microscopy. It is well-known that the dog has contributed to some advances in applied medicine in humans. For this reason, this research encourages researchers in both veterinary medicine and human medicine to explore other diagnostic tools such as scanning electron microscopy.

SEM is a tool useful for determining morphological alterations in the membrane of the RBC as indicative of diseases but is not fully accessible in veterinary medicine for economic reasons and knowledge when interpreting the results because the blood circulation occurs in 3D and, it is how the cells look in SEM; whereas the evaluating a smear with MO, when “squashing” the cells change the dimension. Thus, the interpretation differs remarkably, the first exponent is that in SEM the nucleus is not seen and in the case of white blood cells (WBC), the interpretation is quite complicated by not having more than the morphological aspect of the membrane. Scanning electron microscopy has been shown to be a useful tool for detecting morphological changes in aged dogs. Therefore, this study lays the groundwork for continuing the use of electron microscopy as a diagnostic tool in veterinary medicine since it is important to know the effects of some illnesses and aging on the morphology of RBC. This would help clinicians to consolidate patients' reports and to detect diseases in time, as well as to improve their treatment.

## Data availability statement

The original contributions presented in the study are included in the article/Supplementary material, further inquiries can be directed to the corresponding author.

## Ethics statement

The animal study was reviewed and approved by Ethics regulations for the use of animals in teaching and research at the Autonomous University of Aguascalientes Code: DI-PL-NO-37. Written informed consent was obtained from the owners for the participation of their animals in this study.

## Author contributions

AM-N and AG-B conceived this study, participated in its design, performed coordination, and helped draft the manuscript. TQ-T, AV-F, and MC-R made significant contributions to the conception, design, and analysis of the results. AM-N also carried out laboratory analysis and statistical analysis. All authors contributed to manuscript revision, read, and approved the submitted version.

## Funding

The Autonomous University of Aguascalientes approved and granted funding for TQ-T (Project PIP/SA15–3) for AG-B (PIB19-3, and Especial Resource UAA Research), which was used in the acquisition of materials and reagents necessary to obtain and process blood samples during the development of this study.

## Conflict of interest

The authors declare that the research was conducted in the absence of any commercial or financial relationships that could be construed as a potential conflict of interest.

## Publisher's note

All claims expressed in this article are solely those of the authors and do not necessarily represent those of their affiliated organizations, or those of the publisher, the editors and the reviewers. Any product that may be evaluated in this article, or claim that may be made by its manufacturer, is not guaranteed or endorsed by the publisher.
