# Supplementary material for: Red blood cells morphology and morphometry in adult, senior, and geriatricians dogs by optical and scanning electron microscopy
Source: Front Vet Sci. 2022 Nov 10;9:998438. doi: 10.3389/fvets.2022.998438 (PMC9685804; doi:10.3389/fvets.2022.998438)
Supplement: Supplementary file 1 [file Data_Sheet_1.PDF]

Screening of apparently healthy senior and geriatric dogs: Owner-questionnaire (Appendix S1).

1) How would you describe the general health of your dog? (Mark the correct answer).

☐Very good ☐Good ☐Fair ☐Bad ☐Very bad

2) Living environment (tick the correct answer).

☐City ☐Field

3) Lifestyle (tick one or more correct answers).

☐ Daily walks
☐ Permanently outdoors
☐ Can decide him/herself to go outside
☐ Regularly released in garden
☐ Only inside the house
☐ Other:

4) Your dog is?

☐ Active ☐ Passive (quiet / little activity)

Has this changed compared to a few years ago? ☐No ☐Yes

If your answer is affirmative, specify what changes have you observed?

5) How many walks do you take with your dog and what is the average distance?

6) Have you noticed any behavioral changes?

☐No ☐Yes, describe.

- How often does your dog pace up and down, walk in circles and/or wander with no direction or purpose?
  ☐ Never ☐ 1x/month ☐ 1x/week ☐ 1x/day ☐ >1x/day
- How often does your dog stare blankly at the walls or floor?
  ☐ Never ☐ 1x/month ☐ 1x/week ☐ 1x/day ☐ >1x/day
- How often does your dog get stuck behind objects and is unable to get around?
  ☐ Never ☐ 1x/month ☐ 1x/week ☐ 1x/day ☐ >1x/day
- How often does your dog fail to recognise familiar people or pets?
  ☐ Never ☐ 1x/month ☐ 1x/week ☐ 1x/day ☐ >1x/day
- How often does your dog walk into walls or doors?
  ☐ Never ☐ 1x/month ☐ 1x/week ☐ 1x/day ☐ >1x/day
- How often does your dog walk away while, or avoid, being patted?
  ☐ Never ☐ 1x/month ☐ 1x/week ☐ 1x/day ☐ >1x/day
- How often does your dog have difficulty finding food dropped on the floor?
  ☐ Never ☐ 1-30% of times ☐ 31-60% of times ☐ 61-99% of times ☐ Always

For the following questions: in case the problem does not present itself, please tick ‘The same’

- Compared with 6 months ago, does your dog now pace up and down, walk in circles and/or wander with no direction or purpose
  ☐ Much less ☐ Slightly less ☐ The same ☐ Slightly more ☐ Much more
- Compared with 6 months ago, is the amount of time your dog spends active
  ☐ Much more ☐Slightly more ☐ The same ☐ Slightly less ☐ Much less
- Compared with 6 months ago, does your dog now stare blankly at the walls or floor

☐ Much less ☐ Slightly less ☐ The same ☐ Slightly more ☐ Much more

- Compared with 6 months ago, does your dog urinate or defecate in an area it has previously kept clean

  ☐ Much less ☐ Slightly less ☐ The same ☐ Slightly more ☐ Much more
- Compared with 6 months ago, does your dog have difficulty finding food dropped on the floor (Score x2)

  ☐ Much less ☐ Slightly less ☐ The same ☐ Slightly more ☐ Much more
- Compare with 6 months ago, does your dog fail to recognise familiar people or pets? (Score x3)

  ☐ Much less ☐ Slightly less ☐ The same ☐ Slightly more ☐ Much more

To be filled out by veterinarian: total Canine Cognitive Dysfunction Rate =………

(Questionnaire based on Salvin HE, McGreevy PD, Sachdev PS, et al. The canine cognitive dysfunction rating scale (CCDR): A data-driven and ecologically relevant assessment tool. Vet J 2011;188:331)

7) Are there any other pets in the household? If so, which one and how many?

☐No ☐Yes, explain

8) Is your dog eating normally? Less or more than previously? If abnormal, please describe.

9) Is your dog drinking normally? Less or more than previously? If abnormal, please describe.

10) Is urination normal? Is the position normal? Do you have the impression it is difficult or painful for your animal?

11) Is defecation normal? Is the position normal? Do you have the impression that it is difficult or painful for your animal?

12) Has your dog recently vomited? ☐No ☐Yes

When and how often?

Description:

Was any treatment given, which one?

13) Has your dog recently suffered from diarrhea? ☐No ☐Yes

When and how often?

Description:

Was any treatment given, which one?

14) Has your dog presented coughing, sneezing or nasal discharge? ☐No ☐Yes

When and how often?

Description: ☐Productive (phlegm or mucus). ☐ At rest.

☐Not productive (dry). ☐ During exercise.

Was any treatment given, which one?

15) Do you have the impression your dog fatigues faster during (moderate) exercise?

☐No ☐Yes, please describe.

16) Does your dog show an accelerated breathing pattern and/or difficult breathing? ☐No ☐Yes

If yes, when? ☐During break and / or ☐During activity

17) What food does your dog receive? (brand + type) you can select more than one answer.
☐ Commercial foods (croquettes):
☐ Kibbles/canned food:
☐ Home-cooked diet:
☐ Raw meat:
What amount?
How many times a day or is it at will (Ad libitum)?
Recent changes in food? ☐No ☐Yes (when?)
Do you receive anything extra (prizes, baits...)? ☐No ☐Yes

18) Is the weight of your dog stable? Please specify
☐No ☐Yes
If the answer is no, specify if you have noticed that it increases or decreases ...

19) Has your dog received any medication in the last two months?
☐No ☐Yes
What product, when, how long?

20) Are vaccination and deworming performed regularly? ☐No ☐Yes
When was the last time?

21) Is your dog treated against fleas, ticks, …? ☐No ☐Yes
When was the last time?

22) Do you brush the teeth of your dog? ☐No ☐Yes
If so, how often?

23) Has your dog ever been outside Aguascalientes? ☐No ☐Yes
Where and when?

24) Does your dog have any important medical history? (illness, surgery, trauma, medication)

25) When was his/her last veterinary visit? How frequently is he/she seen by a veterinarian?

26) Has the dog previously been checked for hypertension of heart murmur?

☐No ☐Yes When?

Which it was the result?

27) Has the dog previously had a blood examination performed?

☐No ☐Yes When?

What test (s)?

Which it was the result?

28) Has an echocardiography previously been performed?

☐No ☐Yes When?

Which it was the result?

(Questionnaire based on Willems, A., Paepe, D., Marynissen, S., Smets, P., Van de Maele, I., Picavet, P., Duchateau, L. and Daminet, S. (2017), Results of Screening of Apparently Healthy Senior and Geriatric Dogs. J Vet Intern Med, 31: 81-92. https://doi.org/10.1111/jvim.14587).

Informed consent

I am the owner or representative of the pet owner and I have the authority to execute this consent, therefore, I give my authorization for the named pet to undergo a clinical examination and take a blood sample (jugular or cephalic vein) to perform laboratory tests (blood chemistry) for free as part of the research project of the MVZ Ana Luisa Montoya Navarrete thesis of the Autonomous University of Aguascalientes. I understand that necessary precautions will be taken to reduce stress and safeguard my pet's health during the procedure.

My signature below indicates that I have read and understand this consent.

Signature: Date:
