# Supplementary material for: Red blood cells morphology and morphometry in adult, senior, and geriatricians dogs by optical and scanning electron microscopy
Source: Front Vet Sci. 2022 Nov 10;9:998438. doi: 10.3389/fvets.2022.998438 (PMC9685804; doi:10.3389/fvets.2022.998438)
Supplement: Supplementary file 2 [file Data_Sheet_2.PDF]

Medical history format
(Appendix S2)

Number:	Date:

OWNER INFORMATION

Owner's name:
Address:
Phone:
Mail:

PATIENT'S DATA

Name:	Date of birth and age:	Sex: H/M	Breed:	Weigth:	Size:

Particular signs:

Vaccination: yes / no<br>Date:	Deparasitation: yes / no<br>Date:	Is your dog spayed? yes / no
Type of food	Fast: yes / no<br>8h 12h	Origin<br>rural / urban

Anamnesis:

T°	Heart rate	Pulse	Mucous	Capillary fill time *seg*
Breathing frequency	Palmopercusión	Tusigen reflex	Swallowing reflex	Body condition
Hydration	Lymphonodes	Abdominal palpation	Attitude	

Observations (eyes, ears, skin and annexes, locomotion, skeletal muscle, nervous, cardiovascular, respiratory, digestive, genitourinary system):

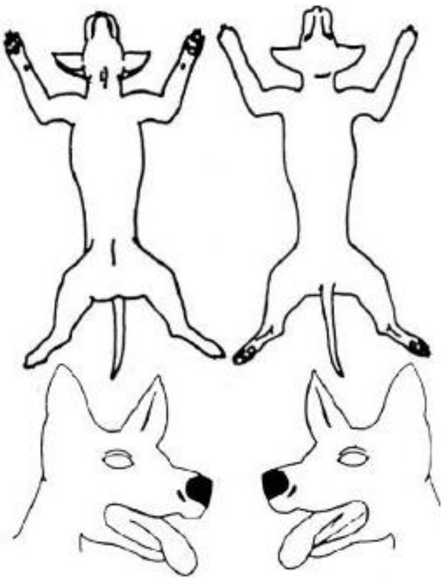

Previous illnesses or procedures:
